# Stacked Transistor Bias Circuit of Class-B Amplifier for Portable Ultrasound Systems

**DOI:** 10.3390/s19235252

**Published:** 2019-11-29

**Authors:** Hojong Choi

**Affiliations:** Department of Medical IT Convergence Engineering, Kumoh National Institute of Technology, 350-27 Gumi-daero, Gumi 39253, Korea; hojongch@kumoh.ac.kr

**Keywords:** stacked transistor bias circuit, class-B amplifier, portable ultrasound system, transducer

## Abstract

The performance of portable ultrasound systems is affected by the excessive heat generated by amplifiers, thereby reducing the sensitivity and resolution of the transducer devices used in ultrasound systems. Therefore, the amplifier needs to generate low amounts of heat to stabilize portable ultrasound systems. To properly control the amplifier, the related bias circuit must provide proper DC bias voltages for long time periods in ultrasound systems. To this end, a stacked transistor bias circuit was proposed to achieve a relatively constant amplifier performance irrespective of temperature variance without any cooling systems as the portable ultrasound system structure is limited. To prove the proposed concept, the performance of the gain and DC current consumption at different experimental times was measured and compared to a developed class-B amplifier with different bias circuits. The amplifier with the stacked transistor bias circuit outperformed with regard to the gain and DC current variance versus time (−0.72 dB and 0.065 A, respectively) compared to the amplifier with a typical resistor divider bias circuit (−5.27 dB and 0.237 A, respectively) after a certain time (5 min). Consequently, the proposed stacked transistor bias circuit is a useful electronic device for portable ultrasound systems with limited structure sizes because of its relatively low gain and DC current variance with respect to time.

## 1. Introduction

Portable ultrasound systems have been widely used for medical applications requiring system portability and capability for immediate diagnosis [1]. Compared to the performance of conventional bench-top ultrasound systems, that of portable ultrasound systems is negatively influenced by excessive heat production due to limited structure size and rapid battery depletion [1]. The heat issue has been addressed by the addition of aluminum heat pipe structures with additional fan systems. To overcome battery issues, solar cell batteries have been used as an additional option; however, they are quite bulky and cannot be used at night [2]. 

The battery issues are caused by the amplifiers, which are the most dominant power-consuming electronic components in portable ultrasound systems. In particular, proper design of the amplifiers is essential to achieve an adequate sensitivity and resolution performance of the system [3,4,5]. To resolve these power-consuming electronic devices, nonlinear amplifiers are one potential candidate [6]. Therefore, nonlinear amplifiers used in portable ultrasound systems could achieve a performance that is less sensitive to temperature and DC power compared to the linear amplifiers used in bench-top ultrasound systems, because nonlinear amplifiers produce a relatively low output waveform with a limited DC power supply [7]. 

The design of amplifiers is quite challenging because there are insufficient simulation library models with temperature model parameters of semiconductor devices such as lateral diffusion metal–oxide–semiconductor field-effect transistors (LDMOSFETs) and power film resistors [8]. Moreover, equivalent circuit models of LDMOSFETs remain under development [9]. Consequently, the design of amplifiers still requires hands-on fabrication, testing procedures, and performance adjustment. There is limited nonlinear amplifier research into several different types of transducers. 

In addition, there is very little research into nonlinear amplifiers used in portable ultrasound systems. Nonlinear amplifier research would be useful for portable ultrasound systems due to the relatively low current or power consumption. For example, a class-D amplifier was developed for 41 kHz high-power piezoelectric loads [10], while a class-E amplifier was developed for an inductive transducer [11]. A class-DE amplifier was developed for piezoelectric transducers used in MRI-compatible high-intensity focus ultrasound therapy [12]. For such systems, the temperature can still be critical for long-term operation. It is for these reasons that the particular bias circuit is proposed for use in nonlinear amplifiers for portable ultrasound systems. 

The bias-voltage stabilizer with a regulator was proposed to reduce unwanted input pulses [13]. The linearizer circuit was developed for high-frequency ultrasound systems to reduce gain deviation [14]. Another linearizer circuit was also developed to reduce gain deviation for ultrasound transducers [15]. However, the stabilizer with regulator and linearizer circuits was intended to be used for a class-A amplifier for a typical bench-top ultrasound system with cooling systems.

For communication systems, stacked transistor bias circuits have been used in low-voltage circuit design [16]. However, this is the first time that this topology has been used in high-voltage circuit design for any type of ultrasound electronics; bench-top ultrasound machines always use large cooling fans and heat-pipe structures to obtain a relatively constant system performance, and thus might not have required such heat-reducing innovations. To apply stacked transistor bias circuits used in high-voltage circuits, the low-voltage transistor cannot be used in the bias circuit, as large DC current and high-voltage input amplitudes pass through the bias circuit, leading to the bias circuit not working properly and causing a heat problem in the primary transistors of the amplifier. The particular stacked transistor bias circuits need to handle those problems together. 

Therefore, a nonlinear amplifier integrated with a stacked transistor bias circuit would be beneficial to obtain constant performance for portable ultrasound systems, which could be less sensitive to other environmental parameters such as temperature and batteries. Additionally, a high-temperature sustainable piezoelectric transducer array was also developed to obtain constant echo signals irrespective of the temperature [17].

**Figure 1:**
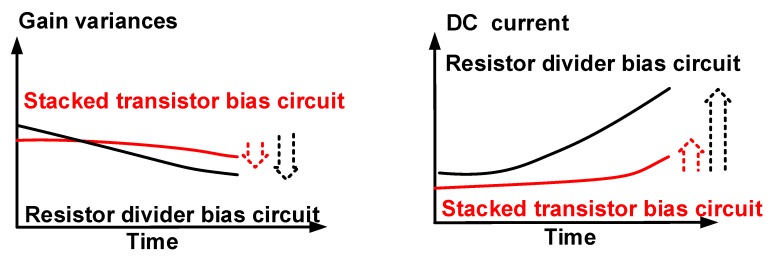
Proposed concept of amplifiers with a stacked transistor bias circuit.

Figure 1 shows the proposed concept for the stacked transistor bias circuit for portable ultrasound systems. Though amplifiers operate constantly inside the enclosed structure of the portable ultrasound system, the amplifier performance needs to be constant, irrespective of the operating time. However, the amplifier performance worsens with increasing time, requiring a cooling fan with heat-pipe structures to cool down the amplifier, resulting in a sustained performance at the expense of high mechanical noise from the cooling systems. Amplifiers that can sustain performance over long time periods are highly desirable. The proposed stacked transistor bias circuit supports stable gain and DC current variance, as described in Figure 1. 

In Section 2, schematic diagrams of the resistor divider and the proposed stacked transistor bias circuits are presented, and the operating mechanisms of the proposed circuits are analyzed. Section 3 presents measured results of the proposed circuits and the pulse-echo response system. Section 4 presents the concluding remarks to this manuscript.

## 2. Materials and Methods 

### 2.1. Fabricated Class-B Amplifiers with Bias Circuits

**Figure 2:**
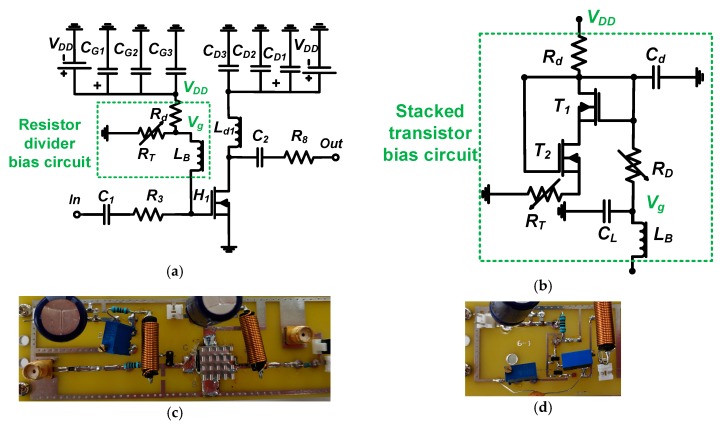
Full schematic diagram of (**a**) single-ended class-B amplifier with resistor divider and (**b**) stacked transistor bias circuits; implemented printed circuit boards of (**c**) single-ended class-B amplifier with resistor divider bias circuit and (**d**) stacked transistor bias circuit only.

Figure 2 presents full schematic diagrams, and images of the implementation, of the class-B amplifier with a resistor divider and stacked transistor bias circuits on printed circuit boards. In Figure 2a, a class-B amplifier of the single-ended type was used because the push−pull class-B amplifier architecture using the transformers is quite sensitive to the transducer, which has a fast detection time due to the echo duration period [3]. The developed amplifiers operate in a relatively high-voltage environment, so one fixed large 1 kΩ resistor (*R_d_*) was used to reduce the DC current despite the DC bias voltages being limited. A 50 Ω output resistor (*R*_8_) was used after the 220 pF capacitors (*C*_2_), and 1 μH choke inductors (*L_B_* and *L*_*d*_1__) were used near the gate and drain sides of the LDMOSFET (PD57006S-E, STMicroelectronics Inc., Geneva, Switzerland) for proper operating frequency ranges. Choke inductors (*L_B_* and *L*_*d*_1__, Bourns Inc., Riverside, CA, USA) were used to minimize DC voltage reduction from the power supply (*V_DD_*), because the maximum AC output voltages are less than the DC input voltage of the power supply (*V_DD_*). The choke inductors work at a relatively high impedance for AC signals and a low impedance for DC signals. To reduce the AC ripple currents produced by the power supply, 220 μF electrolytic capacitors (*C*_*G*_1__ and *C*_*D*_1__, Panasonic North America, Newark, NJ, USA) with additional 100 nF and 1000 pF ceramic capacitors (*C*_*G*_2__ and *C*_*D*_2__, and *C*_*G*_3__ and *C*_*D*_3__ Panasonic North America, Newark, NJ, USA) were used. A 220 pF input DC coupling capacitor (*C*_1_, Vishay Siliconix, Santa Clara, CA, USA) was used because they can block unwanted AC noise signals generated by the digital-to-analog converter. The output DC coupling capacitors (*C*_2_, Vishay Siliconix, Malvern, PA, USA) were used because high-voltage DC signals generated by the DC power supply can affect the image quality of ultrasound systems. 

As shown in Figure 2a, the resistor divider bias circuit is a very simple voltage divider circuit. However, it may not be desirable for use with amplifiers that produce temperature variations caused by high-voltage operations. Performance deviation caused by temperature variance is undesirable because it affects transducers by generating an acoustic power variance [17]. Therefore, temperature-insensitive bias circuits are proposed to minimize performance variation, irrespective of temperature. In Figure 2b, the structures are composed of two LDMOSFETs (*T*_1_ and *T*_2_, SQ2318AES-T1_GE3, Vishay Siliconix, Santa Clara, CA, USA) operating in a current mirror architecture. This architecture provides relatively constant DC bias voltages [16]. A 1 kΩ fixed resistor (*R_d_*, Panasonic North America, Newark, NJ, USA) was used to reduce the DC current from the power supply and two 1 kΩ trimmers (*R_D_* and *R_T_*, Vishay Siliconix, Malvern, PA, USA) generated variable DC bias voltages. A low-pass filter composed of a choke inductor (*L_B_* = 1000 nH, Bourns Inc., Riverside, CA, USA) and a capacitor (*C_L_* = 120 pF, Vishay Siliconix, Santa Clara, CA, USA) was also used to reduce undesirably high input AC voltages coming from the input, and to reduce further possible noise signals from the power supply.

In Figure 2c, the class-B amplifier is of the nonlinear type, which can produce less heat compared to class-A amplifiers. However, because heat can affect the stability of the operation of the amplifier, a 1 cm^2^ heat sink with thermal grease was attached to the top of each LDMOSFET to facilitate heat transfer. In Figure 2d, a stacked transistor bias circuit on the printed circuit board is shown. The printed circuit board was fabricated by the manufacturer (ExpressPCB, Mulino, NY, USA). The next section will describe operating mechanisms of the bias circuits for single-ended class-B amplifiers. The amplifier output caused by temperature variance can affect transducer performance, resulting in an increased sensitivity of the performance of the ultrasound system. 

### 2.2. Equivalent Circuit Analysis of Class-B Amplifier with Bias Circuits

**Figure 3:**
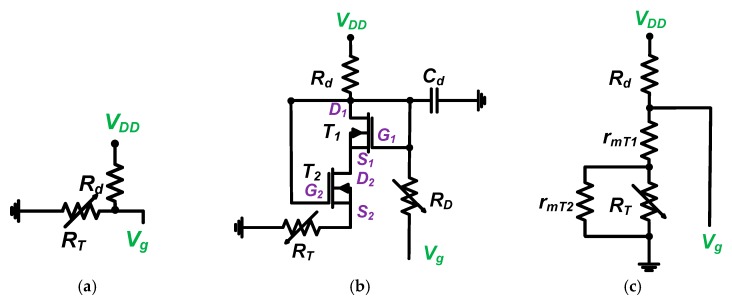
(**a**) Resistor divider bias circuit and (**b**) stacked transistor bias circuit without choke inductor; (**c**) equivalent circuit model of (**b**).

Figure 3 presents an equivalent circuit model of a resistor divider and stacked transistor bias circuits to analyze the DC bias voltage. 

As shown in Figure 3a, the bias voltage of the resistor divider bias circuit can be simply obtained by the ratio of the resistors (*R_d_* and *R_T_*) in Equation (1),

(1) $$V_{g}=\frac{R_{T}}{R_{d}+R_{T}}V_{DD\;}.$$

Figure 3b describes the operating regions of each top and bottom transistor (*T*_1_ and *T*_2_). For the top transistor (*T*_1_), the drain-source voltage (*V*_*DS*_1__) is larger than the gate-source voltage (*V*_*GS*_1__) because the drain (*D*_1_) and gate (*G*_1_) are connected. Therefore, the equivalent circuit model for the top transistor has only drain resistance (*r*_*mT*_1__), as shown in Figure 3c. For the bottom transistor (*T*_2_), the drain-source voltage (*V*_*DS*_2__) is also larger than the gate-source voltage (*V*_*GS*_2__) because the voltage drop through the top transistor (*T*_1_) is small due to the low drain resistance (*r*_*mT*_1__). Therefore, the equivalent circuit model for the top transistor also has only drain resistance (*r*_*mT*_2__), as shown in Figure 3c. Resistor R_D_ is large (1 kΩ), so the voltage drop is negligible. In Equation (2), the drain resistance of the bottom transistor (*r*_*mT*_2__) is much smaller than that of resistor *R_T_*. The equivalent circuit model of the stacked transistor bias circuit can be obtained in Equation (2).

(2) $$V_{g}=\frac{r_{mT_{1}}+(r_{mT_{2}}//R_{T})}{R_{d}+r_{mT_{1}}+(r_{mT_{2}}//R_{T})}V_{d}\approx \frac{r_{mT_{1}}+r_{mT_{2}}}{R_{d}+r_{mT_{1}}+r_{mT_{2}}}V_{d},$$

where *r*_*mT*_1__ and *r*_*mT*_2__ are parasitic drain resistances of the stacked transistors (*T*_1_ and *T*_2_, respectively).

**Figure 4:**
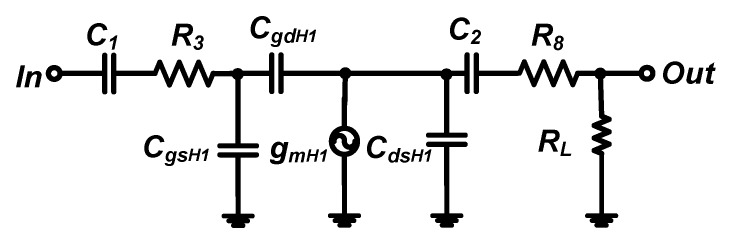
Equivalent circuit model of the class-B amplifier.

Figure 4 shows the equivalent circuit model to estimate the gain of the class-B amplifier. In the equivalent circuit model of the class-B amplifier, the choke inductor (*L*_*d*_1__) has a very large value at the operating frequency (*f_c_*).

The class-B amplifier is a kind of common-source type of amplifier [16]. The gain of the class-B amplifier can be expressed by Equations (3)–(5).

(3) $$Gain=\frac{-G_{mH_{1}}\left(R_{8}//\frac{1}{j2\pi f_{c}C_{2}}\right)}{\left(1+j\frac{f_{c}}{f_{in}}\right)\left(1+j\frac{f_{c}}{f_{out}}\right)},$$

(4) $$\;f_{in}=\frac{1}{2\pi \left(R_{8}+\frac{1}{2\pi f_{c}}\right)[C_{gsH_{1}}+\left(1+G_{mH_{1}}\left(R_{8}//\frac{1}{j2\pi f_{c}C_{2}}\right)C_{gdH_{1}}\right]},$$

(5) $$\;f_{out}=\frac{1}{2\pi \left(R_{8}//\frac{1}{2\pi f_{c}C_{2}}\right)\left[C_{gdH_{1}}+C_{dsH_{1}}\right]},$$

where *G*_*mH*_1__ is the transconductance of the LDMOSFET (*H*_1_); *C*_*gsH*_1__, *C*_*gdH*_1__, and *C*_*dsH*_1__ are the parasitic gate-source, gate-drain, and drain-source capacitances, respectively; *R_L_* is the load resistance; and *f_in_* and *f_out_* are the input and output pole frequencies, respectively.

The choke inductor (*L*_*d*_1__) operates at a large impedance for high-frequency signals, resulting in the minimization of the voltage reduction from the power supply (*V_DD_*) such that the class-B amplifier operates at the saturation region, because the drain voltage is larger than the gate voltage of the LDMOSFET (*H*_1_). The transconductance of the LDMOSFET (*G*_*mH*_1__) can be defined in Equation (6). Therefore, the gain equations of the class-B amplifier with the resistor divider and stacked transistor bias circuits are the same, except for the different DC bias voltages (*V_g_*), as described in Equations (1) and (2).

(6) $$G_{mH_{1}}=\beta_{n}\left(V_{g}-V_{TH_{n}}\right),$$

where *β_n_* and *V*_*TH_n_*_ are the LDMOSFET gain factor and threshold voltage, respectively.

**Figure 5:**
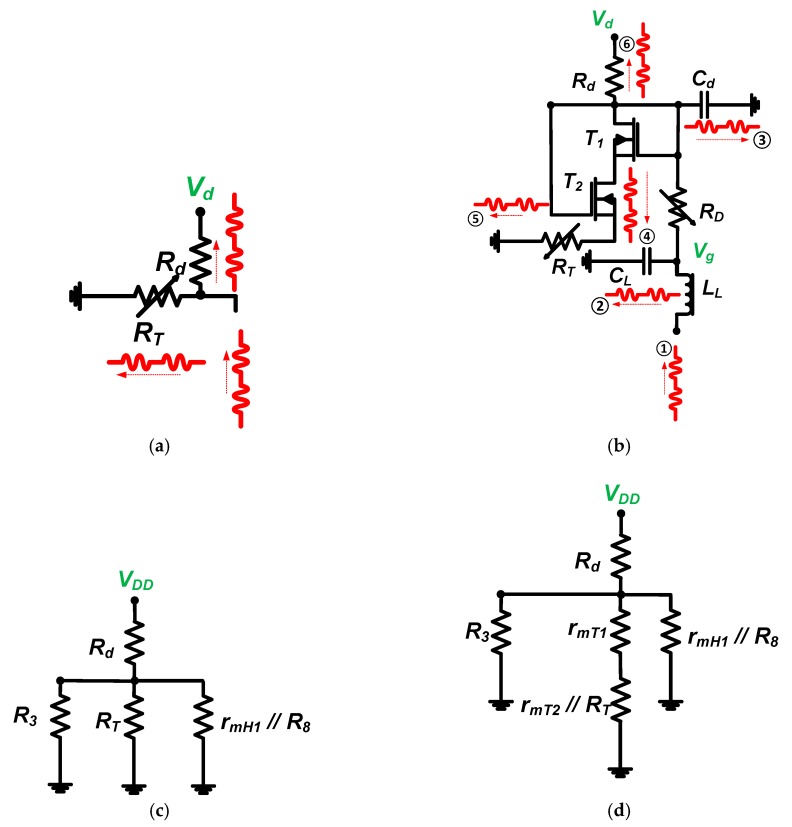
AC signal flow paths for (**a**) resistor divider and (**b**) stacked transistor bias circuits; equivalent circuit model for class-B amplifier with (**c**) resistor divider and (**d**) stacked transistor bias circuits.

The drain resistances (*r*_*mT*_1__ and *r*_*mT*_2__) of stacked transistors are less sensitive to temperature than typical resistors (*R_T_*); we can thus expect the proposed bias circuit to be less susceptible to temperature variance. Figure 5 shows the effects of AC signals on the bias circuits because the AC input signals could affect bias circuit stabilization, in turn affecting the noise level of the echo signals. 

For resistor divider bias circuits, the AC input signal (①) goes to two separate resistors (*R_T_* and *R_d_*, ② and ③), as shown in Figure 5a. For the stacked transistor bias circuit, the AC input signal (①) goes to the first capacitor (*C_L_*, ②) and the second capacitor (*C_d_*, ③). The rest of the AC input signals go to two separate resistors (*R_T_* and *R_d_*, ⑤ and ⑥) through the stacked transistors (*T*_1_ and *T*_2_, ④), as shown in Figure 5b. Therefore, the stacked transistor bias circuit can be useful to suppress unwanted AC input signals in the bias circuits. 

The choke inductors (*L_B_* and *L*_*d*_1__) have negligible values for DC voltages. The equivalent circuit models for DC current analysis are shown in Figure 5c,d. DC currents for the class-B amplifier with a resistor divider bias circuit (*I_DCR_*) and a stacked transistor bias circuit (*I_DCS_*) are presented in Equations (7) and (8), respectively.

(7) $$I_{DCR}=\frac{V_{DD}}{R_{d}+\left(R_{8}//R_{T}//r_{mH_{1}}//R_{8}\right)},$$

(8) $$\;I_{DCS}=\frac{V_{DD}}{R_{d}+\left\{R_{3}//(r_{mT_{1}}+(r_{mT_{2}}//R_{T}))//(r_{mH_{1}}//R_{8}\right\}},$$

where *r*_*mT*_1__ is the parasitic resistance of the LDMOSFET (*H*_1_).

Though the class-B power amplifier with bias circuits was analyzed with the equivalent circuit model, the LDMOSFET simulation library is inaccurate for temperature variance; in the next section, we thus measure the performance of the class-B amplifier to characterize its gain and DC current capabilities [9,18].

## 3. Results and Discussion

### 3.1. Performance of the Class-B Amplifiers with Bias Circuits

**Figure 6:**
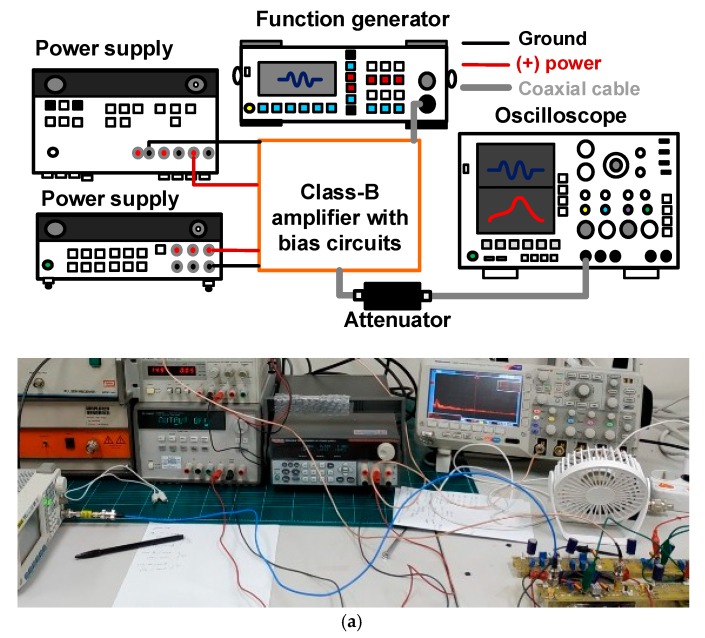

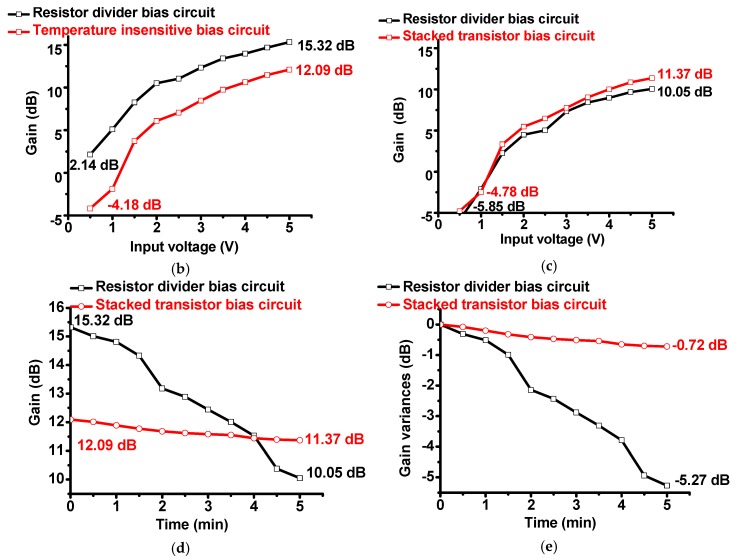
(**a**) Schematic diagram and image of the measurement setup; gain of the class-B amplifier with bias circuits versus input voltages (**b**) at 5 s and (**c**) 5 min, (**d**) and versus time; (**e**) gain variance of the class-B amplifier with bias circuits versus time at 5 V_p-p_ input.

Figure 6a presents the measurement setup of the gain and DC current consumption versus input voltages and input frequencies of the class-B amplifier with bias circuits to characterize the performance. A high and constant gain is desirable for ultrasound systems [19]. Higher output voltages from the amplifiers can increase the sensitivity of the small transducers because of their low sensitivity and constant output voltages; furthermore, a lower current consumption is preferable for portable ultrasound systems with limited battery life and closed structures [20]. As shown in Figure 5a, a 20 MHz, five-cycle sine pulse signal generated by a function generator (DG5701, Rigol Inc., Beijing, China) formed the input for the class-B amplifier with bias circuits. Two power supplies from different manufacturers (2231A-3-30, Keithley Instruments, Cleveland, OH, USA and E3647A, Agilent Technologies, Santa Clara, CA, USA) were the inputs to the gate and drain of the LDMOSFET of the class-B amplifiers. The amplified output voltages were displayed in the oscilloscope (MDO4104C, Tecktronics Inc., Beaverton, OR, USA). The output voltages of the class-B amplifiers were measured to calculate the gain, because the developed ultrasound electronics for transducers were evaluated with respect to the voltage parameters [21]. Though the class-B amplifiers are nonlinear amplifiers, which produce less heat compared to class-A amplifiers, the LDMOSFET cannot be sustained during long time periods under very high temperatures. Therefore, measurement of the class-B amplifiers was performed without constant use of cooling fans and a heat-sink, as shown in Figure 5a. The measurement data were obtained every minute, for 5 min.

In Figure 6b,c, the measured gain of the class-B amplifier with bias circuits versus input voltages is presented at 5 s and 5 min. As shown in Figure 6b, the class-B amplifier with the resistor divider bias circuit (15.32 dB at 5 V_p-p_ input voltage) outperformed that with the stacked transistor bias circuit (12.09 dB at 5 V_p-p_ input voltage). This is because the drain resistances of the stacked transistors (*r*_*Tm*_1__ and *r*_*Tm*_2__) are larger than that of the variable resistor (*R_T_*), as described in Equations (1) and (2). The gain of the amplifiers is dependent on the bias voltages. Therefore, the measured gain of the class-B amplifier with the stacked transistor circuit was lower than that with the resistor divider bias circuit. However, in Figure 5c, the gain of the class-B amplifier with the resistor divider bias circuit changed from 2.14 to −5.85 dB at 0.5 V_p-p_ input voltage, and the gain of the class-B amplifier with the stacked transistor bias circuit changed from −4.18 to −4.78 dB at 0.5 V_p-p_ input voltage. At 5 s, the gain performance of the class-B amplifier with the stacked transistor bias circuit was markedly higher than that with the resistor divider bias circuit. As described in Equations (1) and (2), the stacked transistor bias circuit is less sensitive to temperature variance than the resistor divider bias circuit. Therefore, the gain of the class-B amplifier with the resistor divider bias circuit becomes lower than that with the stacked transistor bias circuit as time passes.

In Figure 6d,e, the measured gain variance of the class-B amplifier with bias circuits versus time is presented, because temperature dependence is an important characteristic for portable ultrasound systems. In Figure 5d, the gain of the class-B amplifier with the resistor divider bias circuit changed from 15.32 to 10.05 dB at 5 V_p-p_ input voltage, and the gain of the class-B amplifier with the stacked transistor bias circuit changed from 12.09 to 11.37 dB at 5 V_p-p_ input voltage. As shown in Figure 5d,e, the gain variance of the class-B amplifier with the stacked transistor bias circuit (−0.72 dB) is much lower compared to that with the resistor divider bias circuit (−5.27 dB). Therefore, we can confirm that the stacked transistor bias circuit assists the class-B amplifier to sustain less gain deviation.

**Figure 7:**
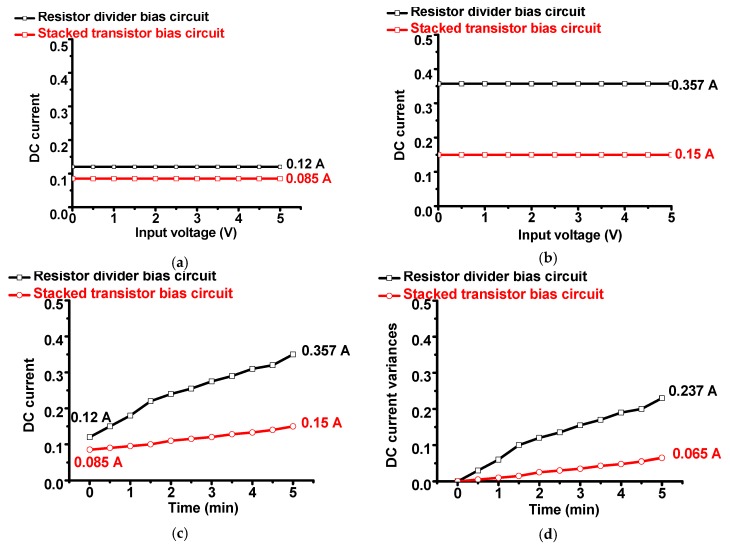
DC current consumption of class-B amplifier with bias circuits versus input voltages (**a**) at 5 s and (**b**) 5 min, (**c**) and versus time; (**d**) DC current consumption variance of class-B amplifier with bias circuits versus time.

Figure 7 presents the DC current consumption and variance of the class-B amplifier with the resistor divider and stacked transistor bias circuits. DC current consumption is one of the most important parameters for portable ultrasound systems because this parameter is related to battery power consumption, as previously mentioned.

In Figure 7a,b, the measured DC current consumption of the class-B amplifier with bias circuits versus input voltage at 1 min intervals over 5 min is presented. In Figure 6a, the measured DC current of the class-B amplifier with the resistor divider bias circuit was slightly higher (0.12 A) than that with the stacked transistor bias circuit (0.085 A) at 5 s. In Figure 6b, the measured DC current of the class-B amplifier with the resistor divider bias circuit was higher (0.357 A) than that with the stacked transistor bias circuit (0.15 A) at 5 min. In Figure 7c,d, the measured DC current consumption and its variance of the class-B amplifier with bias circuits versus time are presented. As shown in Figure 7c, the DC current variance when using the class-B amplifier with both bias circuits worsens as time passes. 

The DC current consumption of the class-B amplifier with both bias circuits was constant irrespective of the input voltage. Therefore, the class-B amplifier with bias circuits was appropriately designed to be applicable to ultrasound systems. Furthermore, the stacked transistor bias circuit can result in a reduced DC current consumption compared to the resistor divider bias circuit with respect to the time period. Consequently, the stacked transistor bias circuit can assist the class-B amplifier to sustain a lower DC current consumption. 

This performance was demonstrated when using the sine waveform with a short pulse period. However, we can expect even worse DC current consumption when using long pulse sine waveforms for pulsed Doppler and color flow imaging, which require a higher power consumption. The gain and DC current consumption variance of the class-B amplifier with the stacked transistor bias circuit were reduced. In addition, we need to apply the designed bias circuit further to evaluate the system performance with the transducer in the next section.

### 3.2. Performance of the Pulse-Echo Responses

**Figure 8:**
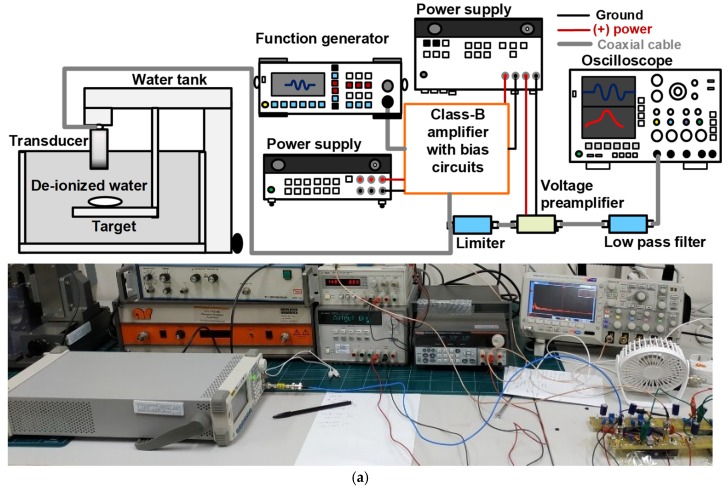

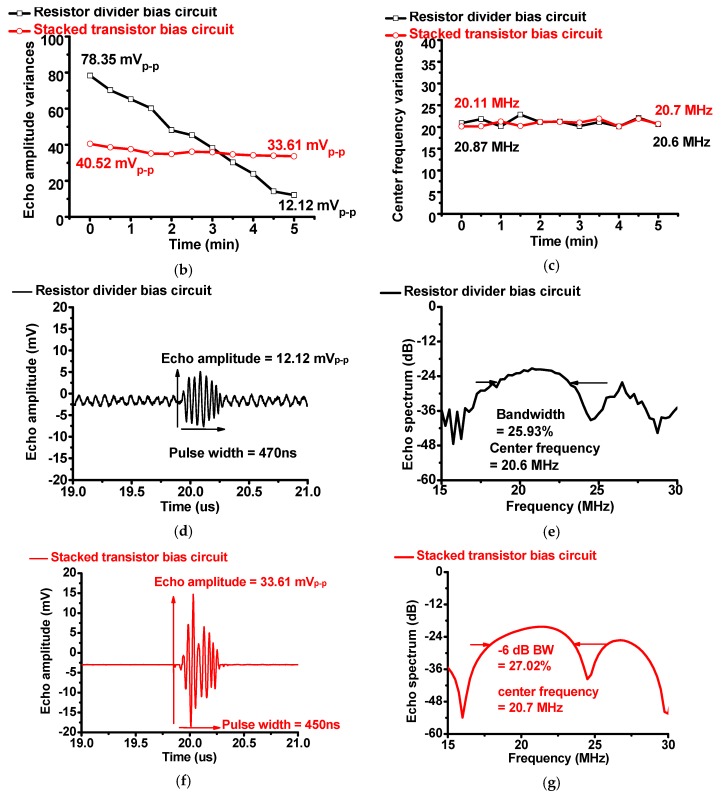
(**a**) Measurement setup schematic diagram and picture of the pulse-echo response using the class-B amplifier with bias circuits; (**b**) echo amplitude and (**c**) spectrum variance when using the class-B amplifier with resistor divider and stacked transistor bias circuits; echo amplitude versus time when using class-B amplifiers with (**d**) resistor divider and (**e**) stacked transistor bias circuits; echo spectrum versus frequency when using class-B amplifiers with (**f**) resistor divider and (**g**) stacked transistor bias circuits.

A-mode pulse-echo responses are fundamental methods for the evaluation of the performance of ultrasound electronics and transducers of ultrasound systems [22,23,24]. Figure 8a presents the measurement setup for the designed class-B amplifier with bias circuit performance. A five-cycle, 5 V_p-p_ pulsed sine waveform generated from the function generator (DG5701) was the input of the class-B amplifier with each bias circuit. While the pulse sine waveform amplified by the designed class-B amplifier with bias circuits was sent to the transducer, the discharged high-voltage pulsed sine waveform was blocked by the limiter to protect the voltage preamplifier [25]. The limiter is a resistor-diode limiter, composed of a 50 Ω power film resistor shunt with a cross-coupled diode.

A reflected echo signal was sent to the limiter, which was then amplified by the voltage preamplifier, and passed through a 90 MHz low-pass filter (Crystek Corporation, Fort Myers, FL, USA) into the oscilloscope (MDO4104C). The low-pass filter was also used to reduce fourth or higher harmonic distortions of echo signals, because nonlinear amplifiers suffer from higher harmonic distortion and noise compared to linear amplifiers. The echo signal received from the transducer in the time and frequency domains was directly displayed in the oscilloscope to obtain the echo amplitude and spectrum. The measured spectrum data were analyzed based on the bandwidth and center frequency of the echo signals. In addition, these measurements needed to be evaluated with respect to the temperature so that the performance could be obtained with respect to the time period.

Figure 8b,c present the echo amplitude and center frequency variance with respect to the time period without cooling systems. As shown in Figure 8b, the echo amplitude when using the class-B amplifier with the resistor divider bias circuit changed from 78.35 to 12.12 mV_p-p,_ and the echo amplitude when using the class-B amplifier with the stacked transistor bias circuit changed from 40.25 to 33.61 mV_p-p_. As shown in Figure 7c, the center frequencies when using class-B amplifiers with the resistor divider and stacked transistor bias circuits were close to 20 MHz. Figure 8d–g present the measured echo signal amplitude and spectrum in the time and frequency domains when using class-B amplifiers with the resistor divider and stacked transistor bias circuits, respectively. These measurement data were obtained at 5 min to check the temperature dependence effects when using class-B amplifiers with bias circuits. The echo amplitude when using the stacked transistor bias circuit (33.61 mV_p-p_) was higher than that when using the resistor divider bias circuit (12.12 mV_p-p_). However, the center frequencies and −6 dB bandwidths when using both bias circuits were similar (20.6 MHz and 25.93% for the resistor divider bias circuit and 20.7 MHz and 27.02% for the stacked transistor bias circuit, respectively). Therefore, we can confirm that stacked transistor bias circuits can assist the class-B amplifier to sustain relatively constant echo signal amplitudes with respect to temperature variance. Table 1 shows the performance comparison of the developed amplifier circuit with class-D, class-E, and class-DE amplifiers except class-A amplifiers, because class-A amplifiers are not suitable for portable ultrasound systems, due to the high power consumption even though the amplifier circuits are used for different transducer types with different conditions such as operating frequency and time period. 

## 4. Conclusions

Portable ultrasound systems typically suffer from limited battery life, excessive heat generation, and limited available numbers of piezoelectric array transducers compared to conventional bench-top ultrasound systems. Some of the most powerful yet power-consuming resources are amplifiers in portable ultrasound systems. Due to the limited structure size of portable ultrasound systems, excessive heat reduces their performance over longer time periods and limits their widespread use for a variety of applications. Therefore, a stacked transistor bias circuit was proposed to provide relatively constant amplifier performance for a constant echo signal of the transducers used in ultrasound systems with less usage of the cooling systems. 

To characterize the performance of the class-B amplifier with bias circuits, gain and DC current consumption were measured over different time periods without cooling fans. At 5 s, the measured gain of the class-B amplifier with the stacked transistor bias circuit (12.09 dB) was lower than that with the resistor bias circuit (15.32 dB), and the measured DC current consumption of the class-B amplifier with the stacked transistor bias circuit was lower (0.085 A) than that with the resistor bias circuit (0.12 A). However, at 5 min, the measured gain of the class-B amplifier with the stacked transistor bias circuit (11.37 dB) was higher than that with the resistor bias circuit (10.05 dB), and the measured DC current consumption of the class-B amplifier with the stacked transistor bias circuit was markedly lower (0.15 A) than that with the resistor bias circuit (0.357 A). Therefore, the stacked transistor bias circuit can sustain a lower gain reduction and better DC current consumption than the resistor divider bias circuit for the class-B amplifier.

To evaluate further capabilities of the proposed idea, the standard evaluation method for ultrasound components was performed. The echo signal amplitude and spectrum when using the resistor divider bias circuit changed from 78.35 to 12.12 mV_p-p_. However, the echo signal amplitude and spectrum when using the stacked transistor bias circuit changed from 40.52 to 33.61 mV_p-p_. The novel stacked transistor bias circuit showed less echo signal performance variance over the time period. Therefore, the stacked transistor bias circuit could be one potential candidate for limiting heat generation in portable ultrasound systems with limited structure size.

## Figures and Tables

**Table 1:** Summarized performances of (**a**) class-D amplifier [10], (**b**) class-E amplifier [12], (**c**) class-DE amplifier [11], and (**d**) the developed amplifier.

Parameters	(a)	(b)	(c)	(d)
Operating frequency	41 kHz	42.05 kHz	973 kHz	25 MHz
Output voltage	60 V	58.76 V	20 V	20.12 V
Power consumption	39.7 W	0.133 W	0.83 W	3.75 W
